# Correlation of intestinal microbiota with overweight and obesity in Kazakh school children

**DOI:** 10.1186/1471-2180-12-283

**Published:** 2012-11-28

**Authors:** Peiru Xu, Min Li, Jihong Zhang, Tao Zhang

**Affiliations:** 1 Department of Pediatrics, First Affiliated Hospital of Xinjiang Medical University, 137 Liyushan South Road, Urumqi, Xinjiang Uyghur Autonomous Region 840054, People’s Republic of China; 2 Xinjiang Evidence-based Medicine Research Institute, 137 Liyushan South Road, Urumqi, Xinjiang, Uyghur Autonomous Region 830054, People’s Republic of China; 3 Department of Urology, Second Affiliated Hospital of Xinjiang Medical University, 38 nanhu East Road, Urumqi, Xinjiang Uyghur Autonomous Region, 830063, People’s Republic of China

**Keywords:** Obesity, *Bacteroidetes*, *Firmicutes*, Kazakh

## Abstract

**Background:**

This study sought to investigate a possible correlation between the intestinal microbiota, *Bacteroidetes* and *Firmicutes*, and obesity in Kazakh school children, aged 7–13 (n = 175).

**Results:**

Obese subjects had significantly greater systolic blood pressure, waist and hip circumference, as well as HOMA-IR as compared to normal and overweight participants. In addition, *Bacteroides* copy number and Bact/Firm ratios were significantly lower in the obese group as compared to the normal and overweight groups (*P* < 0.0167). This difference is only significant in girls, but not in boys when stratified by gender. Furthermore, a negative correlation between BMI and *Bacteroidetes* copy number (r = −0.18, *P* = 0.017) as well as Bact/Firm (r = −0.22, *P* = 0.003) was observed.

**Conclusion:**

An association between reduced gut *Bacteroidetes* and Bact/Firm ratio with obesity in female Kazakh children was identified. Further studies are necessary to elucidate the mechanism behind these changes as well as the value of determining their presence for predicting obesity.

## Background

The incidence of obesity is increasing in an exponential manner worldwide and cannot be explained by genetic factors alone. Thus, a potential role for environmental factors (e.g., life style, geographical environment, feeding patterns etc.) has been increasingly explored in the pathogenesis of obesity. Recent evidence has revealed the influence of gut microbiota on the regulation of nutrient absorption, metabolism, and immune response [1,2].

In vivo studies have demonstrated that an imbalance in gut microbiota might play an important role in the pathogenesis of obesity [3-7]. Specifically, Ley et al. [8] observed reduced *Bacteroidetes* and increased *Firmicutes* levels in obese (ob/ob) mice. However, the correlation between an imbalance in gut microbiota and obesity varies among different human populations. Whereas some studies have observed reduced *Bacteroidetes* in obese subjects [4,6,9], others have reported opposite results [10,11]. In addition, Duncan et al. [12] found no marked difference in *Bacteroidetes* levels between obese and normal weight subjects.

*Bacteroidetes* are nonendospore-forming anaerobes with bile resistance, accounting for more than 25% of gastrointestinal microbiota [13-15]. Because they absorb and metabolize polysaccharides [3] as well as promote the absorption of monosaccharides [16,17], their metabolic activities may be related to obesity occurrence [18]. In addition, *Bacteroidetes* help maintain the balance in gastrointestinal microbiota [17,19]. Although the compositions of gastrointestinal microbiota have been identified, the ways in which these bacteria function remain poorly understood.

Because many of the previous studies linking gut microbiota with obesity have been performed in small groups, which may account for the discrepant results reported, this case-controlled study sought to investigate the role of gut microbiota and obesity in Kazakh children with more participants. The Kazakh people represent a minority in the Xinjiang Province of China. Most Kazakhs live in farming communities and pastoral areas that are underdeveloped, and the incidence of overweight and obesity is relatively high [20,21]. Previous studies have confirmed that the occurrence of obesity in Kazakh preschool children was related to genetic factors [22,23]. In this study, real-time fluorescence quantitative PCR (Q-PCR) was employed to detect *Bacteroidetes* and *Firmicutes* levels and their possible correlation with obesity.

## Methods

### Study participants and study design

This case-controlled study was carried out in the Yili Kazakh Autonomous Prefecture of China. Kazakh children (ages 7–13 y) were recruited from 14 schools within two Counties (Yining and Altay Counties), 5 towns (Yining, Gongliu, Xinyuan, Burqin, and Fuyun) and three villages. Informed consent was obtained from the guardians for all study participants, and children were willing to participant in this study. This study was approved by the Ethics Committee of the First Affiliated Hospital of Xinjiang Medical University (Xinjiang,China).

The following exclusion criteria were applied to select the study participants: (1) children aged <7 y or >13 y; (2) use of antibiotics 2 weeks prior to fecal sample collection as they could alter the gastrointestinal microbiota [24]; (3) the presence of stress (e.g., trauma, severe infection, etc.) 2 weeks prior to fecal sample collection; (4) the presence of gastrointestinal symptoms, including abdominal pain, constipation or diarrhea; and (5) a polio vaccination within one month, which may alter gut microbiota levels by the induced immune response to the vaccine.

A total of 5360 children aged 7–13 y were invited to participate in the study. Fecal specimens were collected from 244 children; 69 subjects were excluded based on the exclusion criteria. Thus, analysis was performed in 175 children.

### Measurements and sample collection

After physical examination, study participants meeting the inclusion criteria were recruited, and informed consent was obtained prior to initiation of the study. In the morning, fasting venous blood samples were collected from the participants by the nurses of the Department of Pediatrics. After incubation at room temperature for 30 min, the serum was collected by centrifugation at 3000 r/min for 15 min and separated into aliquots to analyze fasting plasma glucose (FPG), lipid (triglyceride [TG], total cholesterol [TC], high density lipoprotein [HDL], low density lipoprotein [LDL]), and insulin levels using 7060 Automatic Analyzer (HITACHI, Tokyo, Japan). Homeostasis model assessment of insulin resistance (HOMA-IR) was employed to evaluate the degree of insulin resistance [25] and calculated as follows: HOMA-IR = (FPG × FIN)/22.5.

Body weight and height were measured, and body mass index (BMI) was calculated for each participant. Specifically, the children stood straight with their legs close together and arms hanging naturally. Hip circumference was measured along the greater trochanter (accuracy: 0.1 cm). The criteria for overweight/obesity were developed by the Institute of Child and Adolescent Health of Beijing University for Chinese school-age children and adolescents according to BMI [26], which is specific for age and gender. As shown in Table 1, 84 were diagnosed with overweight/obesity (62 with overweight; 22 with obesity), and the mean age was 9.82 ± 1.96 y, and 91 children had normal BMI with a mean age of 9.92 ± 1.98 y.

**Table 1:** S**equences of primers**

**Primer Name**	**Sequence (5’-3’)**	**Tm (°C)**	**Target length**
Firm-primer-F	GTCAGCTCGTGTCGTGA	60°C	178 bp
Firm-primer-R	CCATTGTAKYACGTGTGT	60°C	
Firm-probe	VIC-GTCAANTCATCATGCC-MGBNFQ	65°C	
Bact-primer-F	AGCAGCCGCGGTAAT	60°C	183 bp
Bact-primer-R	CTAHGCATTTCACCGCTA	60°C	
Bact-probe	FAM-CCCTTTAAACCC-MGBNFQ	65°C	

Stool collection boxes were given to each study participant with instructions on proper collection. Fresh feces were collected in the early morning. In the event that the children did not defecate in the early morning, feces were collected at any time of the morning. After collection, the fecal specimens were sent to the physical examination room and stored at −20°C.

### Real-time quantitative PCR (Q-PCR)

Total DNA was extracted from the gut microbiota isolated from the fecal samples. Specifically, the samples were thawed, and total DNA was extracted from 0.2-0.4 g of the feces using a rapid DNA extraction kit (Beijing BioTeke Corporation, Beijing, China). Isolated DNA was then stored at −20°C until subsequent use in Q-PCR.

To prepare the DNA standards, a sequence with 483 bp in length was prepared and inserted into the PCR®-Blunt II TOPO® vector (Invitrogen, USA). To generate the standard curve, the absolute number of template was 10^10^/μL. The following serial dilutions of the original solution were used to generate the standard curve: 10^8^/μL, 10^7^/μL, 10^6^/μL, 10^5^/μL, 10^4^/μL and 10^3^/μL. The standard curves were obtained using the ABI 7500 Fast Q-PCR detecting system (Applied Biosystem, USA) and 7000 System SDS Software for qPCR.

To determine the absolute number of *Bacteroidetes* and *Firmicutes* in the gut microbiota, primers and probes (Invitrogen, Grand Island, NY) for the conservative sequence of the 16S rRNA genes of both strains were synthesized according to those described previously (Table 1) [27-31] along with the Platinum® *Taq* DNA polymerase (Invitrogen). PCR reactions were denatured at 95°C for 2 min followed by 45 cycles of 95°C for 15 s and 60°C for 1 min.

### Statistical analysis

Data were presented as means ± standard deviations (mean ± SD) for continuous data and n (%) for categorical data. Differences between groups were compared using one-way ANOVA with a post-hoc Bonferroni test for continuous data or Kruskal-Wallis with Mann–Whitney U test when the data were not normally distributed. The Pearson chi-square test was used for categorical data. Furthermore, Spearman’s correlation analysis was used to identify the correlation of BMI with levels of *Bacteroidetes* and *Firmicutes*. All statistical assessments were two-tailed and *P*-values <0.05 were considered statistically significant. Statistical analyses were performed using SPSS 15.0 statistics software (SPSS Inc, Chicago, IL, USA).

## Results

A total of 175 subjects (87 boys and 88 girls) with a mean age of 9.87 y (SD = 1.97) were enrolled for evaluation. Subjects were grouped into the normal, overweight, or obese groups based upon their BMI. As shown in Table 2, demographic information, clinical characteristics, and the presence of *Bacteroidetes* and *Firmicutes* are shown for each group. Among the groups, significant differences in BMI, SBP, DBP, waist and hip circumference, insulin, and HOMA-IR levels were noted (all *P* < 0.05). Obese subjects had significantly greater SBP, waist and hip circumference, as well as HOMA-IR as compared to normal and overweight participants (*P* < 0.05). In addition, significant differences in DBP and *Bacteroidetes* were observed between the obese and normal groups.

**Table 2:** Subjects’ demographics, characteristics and microbe microbiota data by group

**Variables**	**Total**	**Normal group**	**Overweight group**	**Obesity group**	**P-values**
	**(n = 175)**	**(n = 91)**	**(n = 62)**	**(n = 22)**	
Age (y)	9.87 ± 1.97	9.92 ± 1.98	9.65 ± 1.87	10.32 ± 2.19	0.368
Sex					0.906
Boys	87 (49.7)	45 (49.5)	30 (48.4)	12 (54.5)	
Girls	88 (50.3)	46 (50.5)	32 (51.6)	10 (45.5)	
BMI, Kg/m^2^	18.87 ± 3.45	16.53 ± 1.69	20.14 ± 1.83^†^	24.94 ± 3.11^†‡^	<0.001^*^
SBP, mmHg	97.66 ± 14.93	94.06 ± 12.68	98.34 ± 13.21	110.64 ± 20.45^†‡^	<0.001^*^
DBP, mmHg	62.16 ± 9.15	60.38 ± 8.1	63.07 ± 9.15	66.93 ± 11.39^†^	0.005^*^
Waist, cm	63 ± 8.7	58.27 ± 4.91	65.08 ± 6.75^†^	76.72 ± 9.22^†‡^	<0.001^*^
Hip, cm	74.48 ± 9.98	70.26 ± 6.65	76.04 ± 8.7^†^	87.52 ± 12.41^†‡^	<0.001^*^
FPG, mmol/L	4.81 ± 0.84	4.88 ± 1.03	4.73 ± 0.57	4.8 ± 0.61	0.569
Triglyceride, mmol/L	1.21 ± 0.53	1.14 ± 0.47	1.27 ± 0.58	1.36 ± 0.55	0.194
Cholesterol, mmol/L	3.67 ± 0.71	3.73 ± 0.71	3.65 ± 0.68	3.52 ± 0.81	0.424
HDL, mmol/L	1.38 ± 0.51	1.35 ± 0.48	1.38 ± 0.56	1.53 ± 0.46	0.206
LDL, mmol/L	1.58 ± 0.43	1.57 ± 0.45	1.58 ± 0.37	1.62 ± 0.48	0.885
Insulin, mmol/L	6.55 ± 3.74	6.1 ± 3.47	6.21 ± 3.28	9.29 ± 4.86^‡^	0.006^*^
HOMA-IR	1.42 ± 0.87	1.34 ± 0.83	1.33 ± 0.76	1.99 ± 1.14^†‡^	0.016^*^
*Bacteroidetes* × 10^7^copies/μL	1.31 ± 1.94	1.5 ± 2.2	1.37 ± 1.77	0.33 ± 0.47^†^	0.002^*^
*Firmicutes* × 10^7^copies/μL	2.58 ± 4.52	2.43 ± 4.53	2.05 ± 3.01	4.7 ± 7.01	0.628
Bact/Firm	0.98 ± 0.71	1.06 ± 0.62	1.03 ± 0.82	0.48 ± 0.52^†‡^	<0.001^*^

(N = 175).

Data were presented as mean ± SD for continuous data and n(%) for categorical data.

Abbreviations: BMI, body mass index; SBP, systolic blood pressure; DBP, diastolic blood pressure; FPG, fasting plasma glucose; HDL, high density lipoprotein; LDL, low density lipoprotein; Bact/Firm, ratio of *Bacteroidetes* to *Firmicutes*.

^*^P < 0.05, indicated significant differences among groups.

^†,‡^*P* < 0.0167, indicated significant differences as compared with the ^†^normal and ^‡^overweight groups.

The copy number of *Bacteroidetes* and *Firmicutes* were also determined and compared among the groups. Significant differences in *Bacteroidetes* copy number and Bact/Firm ratio among the groups were identified (*P* < 0.002 and *P* < 0.001, respectively; Table 3). No significant changes in *Firmicutes* numbers were noted. Spearman’s correlation analysis revealed a negative correlation between *Bacteroidetes* levels and increased BMI (r = −0.18, *P* = 0.017). A negative correlation between Bact/Firm and BMI was also noted (r = −0.22, *P* = 0.003).

Gender differences were observed in *Bacteroidetes* copy number in children of normal weight. Specifically, girls of a normal weight had significantly higher *Bacteroidetes* levels than boys of normal weight (*P* < 0.05; Table 3). Further stratification of bacterial copy number by gender revealed significantly higher *Bacteroidetes* levels in girls of normal weight compared to obese girls (*P* = 0.002); there was no difference in *Bacteroidetes* levels between normal and obese boys.

**Table 3:** **Univariate analysis of the association of *Bacteroidetes *and *Firmicutes *with BMI levels by gender**

**Variables**	**Total**	**Normal group**	**Overweight group**	**Obesity group**	**P-values**
*Total*	(n = 175)	(n = 91)	(n = 62)	(n = 22)	
*Bacteroidetes* × 10^7^copies/μL	1.31 ± 1.94	1.5 ± 2.2	1.37 ± 1.77	0.33 ± 0.47^†^	0.002^*^
*Firmicutes* × 10^7^copies/μL	2.58 ± 4.52	2.43 ± 4.53	2.05 ± 3.01	4.7 ± 7.01	0.628
Bact/Firm	0.98 ± 0.71	1.06 ± 0.62	1.03 ± 0.82	0.48 ± 0.52^†‡^	<0.001^*^
Boy	(n = 87)	(n = 45)	(n = 30)	(n = 12)	
*Bacteroidetes* × 10^7^copies/μL	1.02 ± 1.53	1.00 ± 1.42^a^	1.30 ± 1.86	0.41 ± 0.56	0.218
*Firmicutes* × 10^7^copies/μL	1.99 ± 3.38	1.71 ± 3.32^a^	1.57 ± 2.04	4.12 ± 5.36	0.170
Bact/Firm	1.06 ± 0.81	1.15 ± 0.72	1.12 ± 0.97	0.59 ± 0.59	0.066
Girl	(n = 88)	(n = 46)	(n = 32)	(n = 10)	
*Bacteroidetes* × 10^7^copies/μL	1.59 ± 2.26	1.99 ± 2.69	1.43 ± 1.70	0.23 ± 0.32^†‡^	0.002*
*Firmicutes* × 10^7^copies/μL	3.17 ± 5.37	3.14 ± 5.41	2.50 ± 3.68	5.39 ± 8.87	0.725
Bact/Firm	0.90 ± 0.58	0.98 ± 0.51	0.94 ± 0.66	0.36 ± 0.43^†‡^	0.003*

Data were presented as mean ± SD; Differences among three groups were compared using Kruskal-Wallis test and between two groups were compared using the Mann–Whitney U test because data were not normally distributed.

^*^P < 0.05, indicated significant differences among three groups.

^†,‡^*P* < 0.0167, indicated significant differences as compared with the ^†^normal and ^‡^overweight groups.

^a^*P* < 0.05, indicated significant differences between boys and girls in normal group. No significant difference between boys and girls were found either in overweight group or in obesity group.

## Discussion

The objective of the present study was to investigate a possible correlation between the intestinal microbiota, *Bacteroidetes* and *Firmicutes*, and obesity in Kazakh school children. Significantly reduced *Bacteroidetes* levels as well as Bact/Firm ratio were observed in the obese group as compared to the normal weight participants, which is similar to previous reports [4,6,9,11]. In addition, a negative correlation between *Bacteroidetes* and Bact/Firm ratio with BMI was observed. These results are consistent with those reported by Ley et al. [4] in which decreased *Bacteroidetes* and increased *Firmicutes* was associated with obesity and increased energy absorption [32].

The Kazakh people are a relatively isolated minority in China and have similarities in living environment and diet, which would likely minimize the difference in the gastrointestinal microbiota among individuals. In the present study, the number of *Bacteroidetes* was markedly lower in obese children than in children with normal weight, resulting in a reduced Bact/Firm ratio. No difference in the Bact/Firm ratio was observed between the overweight and normal groups, which is consistent with that reported by Li et al. [9]. However, as previously stated, discrepant results have been reported. For example, in 98 subjects, including 30 with normal weight, 35 with overweight, and 33 obese individuals, the number of *Bacteroidetes* in overweight subjects was higher than that in the normal group. Furthermore, Duncan et al. [12] found that the number of *Bacteroidetes* in obese subjects was comparable with that in normal weight subjects, and the proportion of *Bacteroidetes* remained unchanged following diet control in obese subjects [33].

The present study also found that the difference in *Bacteroidetes* levels observed between the normal and obese children were mainly contributed by the values obtained in the girls as differences in *Bacteroidetes* levels were observed between normal and obese girls but not boys. This is different from that reported by Mueller et al. [34], who reported a higher *BacteroidetesPrevotella* number in male than in female. A result that may be explained by the age differences between these two studies. In addition, gender differences in the level of *Bacteroidetes* and *Firmicutes* were observed in those participants of normal weight, but not in the overweight and obese groups, which is in agreement with Mueller et al. [34]. While the cause of this difference is unclear, gender differences in iron metabolism [35], which affects the composition of microbiota [36,37], may explain the varying levels of *Bacteroidetes* and *Firmicutes* between normal weight girls and boys observed in this study. More studies with large sample size or more populations are needed to elucidate the specific role of gastrointestinal microbiota in the pathogenesis of obesity as well as the influence of gender on microbiota composition.

Although it is known that obesity is associated with changes in composition as well as function of gut microbiota, the mechanism behind this alteration remains to be elucidated. The influence of gut microbiota on nutrient absorption and metabolism has been suggested as a possible mechanism to explain their possible relation to obesity [16]. Alternatively, altered gut microbiota may alter the exposure to obesogenic and diabetogenic environmental chemicals [38]. Furthermore, altered gut microbiota may increase proinflammatory cytokine secretion, which may be related with the low grade inflammation found in obesity and diabetes [7].

The present study has some limitations. Firstly, two main phyla of bacteria, *Bacteroidetes* and *Firmicutes*, were measured in the feces of Kazakh children; however, specific genus and species were not isolated. Schwiertz et al. [11] reported that the number of *Ruminococcus flavefaciens* in overweight or obese subjects was lower than that in subjects with normal weight. In addition, obese subjects had significantly reduced numbers of *Clostridium leptum* and *Bifidobacterium*. Therefore, specific genus and species will be analyzed in further studies. In addition, the limited amount of DNA obtained from the participant samples prevented the inclusion of 16S sequencing, additional qPCR primer sets, and/or metagenomic shotgun sequencing analyses. Finally, the mechanism by which BMI influences *Bacteroidetes* level or vice versa was not investigated in the present study.

## Conclusion

In summary, this study revealed an significant decrease in the number of *Bacteroidetes* in the feces of obese Kazakh girls; no significant changes in *Firmicutes* numbers were noted. Although the number of study subjects is greater than many previous studies, further studies with larger sample sizes are required to confirm our findings as well as identify the mechanism governing this gender difference in the regulation of intestinal microbiota.

## Competing interest

The authors declare that there is no conflict of interest.

## Authors’ contributions

PX: guarantor of integrity of the entire study, study concepts, definition of intellectual content, manuscript review; ML: guarantor of integrity of the entire study, study design, literature research, clinical studies, data acquisition, statistical analysis, manuscript preparation, manuscript editing; JZ: clinical studies, experimental studies, data acquisition; TZ: data acquisition, data analysis. All authors read and approved the final manuscript.
